# A Study on the Mechanical Properties and Impact-Induced Initiation Characteristics of Brittle PTFE/Al/W Reactive Materials

**DOI:** 10.3390/ma10050452

**Published:** 2017-04-26

**Authors:** Chao Ge, Wubuliaisan Maimaitituersun, Yongxiang Dong, Chao Tian

**Affiliations:** State Key Laboratory of Explosion Science and Technology, Beijing Institute of Technology, Beijing 100081, China; gechao@bit.edu.cn (C.G.); 2120140256@bit.edu.cn (W.M.); 2120150235@bit.edu.cn (C.T.)

**Keywords:** reactive materials, PTFE/Al/W composites, brittle, sensitivity, shear-induced initiation

## Abstract

Polytetrafluoroethylene/aluminum/tungsten (PTFE/Al/W) reactive materials of three different component mass ratios (73.5/26.5/0, 68.8/24.2/7 and 63.6/22.4/14) were studied in this research. Different from the PTFE/Al/W composites published elsewhere, the materials in our research were fabricated under a much lower sintering temperature and for a much shorter duration to achieve a brittle property, which aims to provide more sufficient energy release upon impact. Quasi-static compression tests, dynamic compression tests at room and elevated temperatures, and drop weight tests were conducted to evaluate the mechanical and impact-induced initiation characteristics of the materials. The materials before and after compression tests were observed by a scanning electron microscope to relate the mesoscale structural characteristics to their macro properties. All the three types of materials fail at very low strains during both quasi-static and dynamic compression. The stress-strain curves for quasi-static tests show obvious deviations while that for the dynamic tests consist of only linear-elastic and failure stages typically. The materials were also found to exhibit thermal softening at elevated temperatures and were strain-rate sensitive during dynamic tests, which were compared using dynamic increase factors (DIFs). Drop-weight test results show that the impact-initiation sensitivity increases with the increase of W content due to the brittle mechanical property. The high-speed video sequences and recovered sample residues of the drop-weight tests show that the reaction is initiated at two opposite positions near the edges of the samples, where the shear force concentrates the most intensively, indicating a shear-induced initiation mechanism.

## 1. Introduction

Reactive materials, which are characterized by their exothermic and rapid energy release upon impact with a target or when they are impacted, have been of concern for decades. Generally the reactive materials are composed of two or more non-explosive solids and stay inert until subjected to sufficiently strong mechanical stimulus to undergo fast burning or explosion with release of a high amount of chemical energy in addition to their kinetic energy. Due to these distinctive properties, the reactive materials are considered as potential choices for military applications [1].

Polytetrafluoroethylene filled by aluminum and tungsten particles (PTFE/Al/W) is a typical granular reactive material which is formed by pressing or sintering uniformly mixed metal powders into the fluorine-rich matrix to achieve sufficient strength and insensitivity. The chemical reaction will happen between the Al particles and the PTFE matrix while the main purpose of the introduction of W particles is to improve the density and strength of the composite. Up until now, much research into the formulations and fabrication techniques [2], mechanical properties [3,4,5,6,7], and reaction and energy release [8,9,10] has been conducted and has prompted its improvement. Its application has mainly been focused on reactive fragments [11], reactive material lining [12,13], and explosively-formed compound reactive fragments [14]. Ideal mechanical properties such as high strength, high density and plasticity are desirable characteristics for meeting the launching and machining requirements.

However, under some circumstances, the property of brittleness would be a more desirable characteristic. Early in 2008, Daniel B. Nielson [15] proposed an idea of reactive material enhanced projectiles which aimed to inflict additional damage on what he termed thin-skinned targets. Recently, inspired by the same unique properties, reactive materials have been used to replace the low-density inner core material in the penetrator with enhanced lateral efficiency (PELE) [16]. In these two usages, the housing would be breached and the reactive material or energetic composition contained therein would then expand and disperse into the target structure and be ignited when the projectile is subjected to substantial impact. To some extent, more prominent the brittle property of the reactive material is, more sufficient fragmentation and chemical reaction would result. For this purpose, PTFE/Al/W reactive materials of three different compositions, sintered under a much lower temperature to achieve brittle properties, were fabricated to study its mechanical and impact initiation response in this study.

Previous research by the Split Hopkinson Pressure Bar [17] and Taylor rod-on-anvil tests [18], has broadened our understanding of the impact initiation mechanism of PTFE/Al/W materials. The SHPB and Taylor rod-on-anvil tests were high loading rate methods with the strain rate range of 10^3^~10^4^ s^−1^. Typically, the loading process would last for tens of microseconds. By comparison, the drop-weight apparatus is the most widely used among the many methods of conveying reproducible mechanical stimulus to quantify the impact sensitivities and reaction process of reactive materials, and which are also able to meet the requirement of a relatively low loading strain rate, ~10^2^ s^−1^. The impact and compression process would last for hundreds of microseconds and enables the capture of more details by additional diagnostics [19].

A lot of research on PTFE/Al/W energetic materials has been performed using the standard drop-weight apparatus [4,20,21]. J. Cai et al. carried out a series of drop-weight tests on aluminum jackets confined PTFE/Al/W to understand the high-strain, high-strain-rate flow and failure, the recovered sample residues indicated that the majority of the plastic strain was concentrated in the PTFE polymer [4]. Bin Feng et al. found that the energy level required to initiate the PTFE/Al composite was similar for the quasi-static and drop-weight compression [20]. To overcome the intrinsic oscillation associated with the conventional drop-weight setup, a “soft” drop-weight method of placing a Nitrile O-ring on the top of the impact surface was proposed. The resulting traces exhibited much less oscillation than with the conventional apparatus [21].

Based on the above considerations, in this research, the PTFE/Al/W reactive materials of three different compositions sintered under much lower temperature were studied. Firstly, the quasi-static and dynamic mechanical properties of the three types of materials at room and elevated temperatures were tested to compare the effects of component ratios and temperatures. Then mesosacle structural characteristics of the as received and tested samples were observed by the scanning electron microscope (SEM) to analyze the mechanical and failure response of the materials. Finally, a standard drop-weight apparatus as described above, as well as a high-speed photography system, was used to study the sensitivity and impact-initiation process.

## 2. Experimental

### 2.1. Material Fabrication

The materials studied in this research are three types of pressed and sintered mixtures of PTFE, Al and W powders. The W will not take part in the reaction between the PTFE and Al, and the main purpose of the addition of the W particles is to improve the density and strength of the materials. Thus, in order to achieve the maximum energy release during the reaction, all the three compositions were determined through zero-oxygen-balance between the PTFE and Al with fixing the mass ratio at about 25:9. Table 1 tabulates the wt % mass ratios of the three PTFE/Al/W compositions and the corresponding theoretical material densities (TMD).

**Figure 1:**
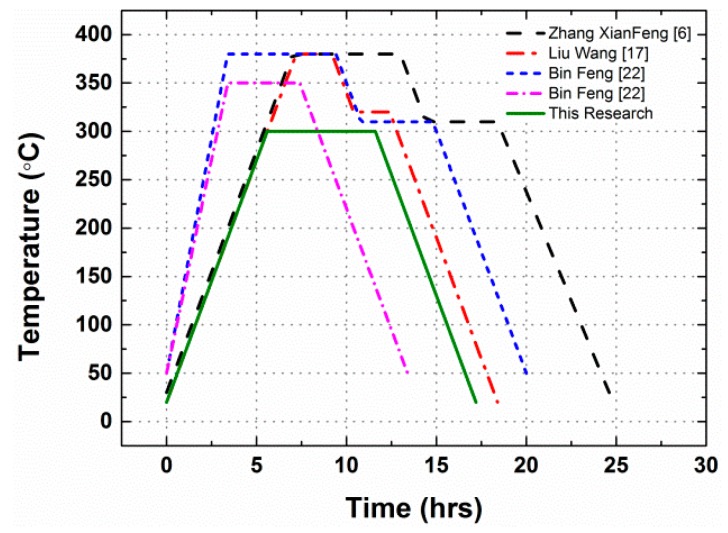
The temperature histories of the sintering cycle.

The preparation process was based on Vasant S. Joshi’s patent [2], but followed a different sintering history. Firstly, powders of Al (JT-4, Hunan Goldsky Aluminum Industry High-Tech Co., Ltd., Changsha, China), PTFE (DuPont PTFE 9002-84-0, type MP 1000, Wilmington, DE, USA) and W (Hunan Zhuzhou Jingzuan Co., Ltd., Zhuzhou, China) were mixed uniformly via a dry mixing process in vacuum at 50 °C for approximately 24 h, before a drying process at 50 °C for ~24 h. The dried mixtures were pressed into a rectangular steel mould to produce rectangular 600 mm × 600 mm × 15 mm samples under the pressure of 70~80 MPa with a dwell time of approximately 10 min. The rectangular samples were then placed into a cold hydrostatic pressing machine to undergo a cold isostatic pressing process from 220 to 250 MPa for another 10 min. Finally, the samples were put into a furnace to undergo a sintering cycle under the protection of argon atmosphere. Different from the sintering processes in published researches, the maximum temperature in our sintering process was much lower while the sintering cycle was much shorter to achieve a more brittle material property [6,17,22]. Specifically, it included heating the pressed samples at a rate of about 50 °C per hour to a final temperature of 300 °C, holding at the final temperature for 6 h, and then cooling to room temperature at the rate of 50 °C per hour. From the 600 mm × 600 mm × 15 mm rectangular materials, samples for the tests were machined. The time history of the sintering cycle is depicted in Figure 1.

### 2.2. Quasi-Static and Dynamic Compression Tests

Quasi-static compression tests of the three types of samples were conducted by using an Instron 5969 (Norwood, MA, USA) servo-hydraulic load frame in a standard laboratory environment (23 ± 2 °C, 30%~40% relative humidity). Dimensions of the samples were Φ10 mm × 10 mm and the loading speed of the crosshead was determined based on the height of the sample to correspond to a nominal strain rate of 10^−3^ s^−1^. Before tests, all contact surfaces of the samples were lubricated to eliminate the effect of friction. In total, nine samples—three for each of the three types of materials—were tested.

The dynamic compression properties of the materials were tested using the Split Hopkinson Pressure Bar (SHPB) system [23]. The valid application of SHPB is based on two fundamental postulates:

(1) One-dimensional elastic stress wave theory is valid in pressure bars; and
(2) Stress and strain rates within the specimen are uniaxial and uniform.

The first postulate can be satisfied by limiting the impact velocity to ensure that the pressure bars deform elastically and by using a proper length–to–diameter ratio of pressure bars and projectile to eliminate the wave dispersion. In this research, a 16-mm-diameter 7075 T6 aluminum Split Hopkinson Pressure Bar (SHPB) system was used to achieve a high signal-to-noise ratio. The length of the striker bar is 200 mm, while that of the incident bar and transmitted bar is 2000 mm. To satisfy the second postulate, the lateral, radial and axial inertia effect and the friction restraint need to be eliminated. Sufficient lubrication of the sample surfaces before they are sandwiched between the incident and transmitted bars can reduce the friction restraint. Davies and Hunter [24] analyzed the error of the stress in the sample caused by the radial and axial inertia to propose a method to determine the optimal dimension of the sample. The corrected stress σ in the sample can be expressed by the following form:

(1) $$\sigma \left(t\right)=\sigma_{m}\left(t\right)+\rho_{s}\left(\frac{{l_{0}}^{2}}{6}-v_{s}\frac{d^{2}}{8}\right)\frac{d^{2}\varepsilon \left(t\right)}{{\mathrm{dt}}^{2}}$$

where $\sigma_{m}$ is the measured stress in the sample, $\rho_{s}$ is the density of the sample, $v_{s}$ is the Poisson’s ratio of the sample while $l_{0}$ and d represent the original length and the diameter of the sample.

In Equation (1), the least error between the measured stress $\sigma_{m}$ and corrected stress σ(t) results when:

(2) $$\frac{{l_{0}}^{2}}{6}-v_{s}\frac{d^{2}}{8}=0$$

or

(3) $$\frac{l_{0}}{d}=\sqrt{\frac{3v_{s}}{4}}$$

Equations (2) and (3) show that the slenderness ratio plays opposite roles on axial and lateral inertia effects. Thus the optimal dimension for the SHPB specimen can be determined by Equation (3) to minimize the inertia effects. The Poisson’s ratio $v_{s}$ of the three types of materials tested in this research can be estimated by the rule of mixtures and the value ranges from 0.37 to 0.39. Thus the optimal slenderness ratio would be between 0.52 and 0.54. Then the dimensions of the samples were determined as Φ11 mm × 6 mm and 60 samples, in total, with 20 for each type of materials, were tested. Considering the low impedance and brittle nature of the material, a slowly rising incident pulse is preferred to a pulse that rises steeply, in order to minimize the effects of dispersion and allow the sample to achieve early dynamic stress equilibrium. Thus polyurea shims with a diameter of 12 mm and varied thicknesses were placed between the striker bar and the incident bar to shape the incident pulse to achieve stress equilibrium [25].

The high-rate characterization of the PTFE/Al/W composites at elevated temperatures was conducted by employing a furnace. To reduce the effect of temperature gradient in the bars, only the specimens were exposed to the temperature environment. Both the incident and transmitted bars were initially separated from the hot specimen. The bar ends were moved into contact with the specimens only shortly before the stress-wave loading. The high-rate compressive response of PTFE/Al/W was characterized at four elevated temperatures, 20 °C, 100 °C, 150 °C and 200 °C at a common strain rate of ~4000 s^−^^1^.

### 2.3. Drop-Weight Test

**Figure 2:**
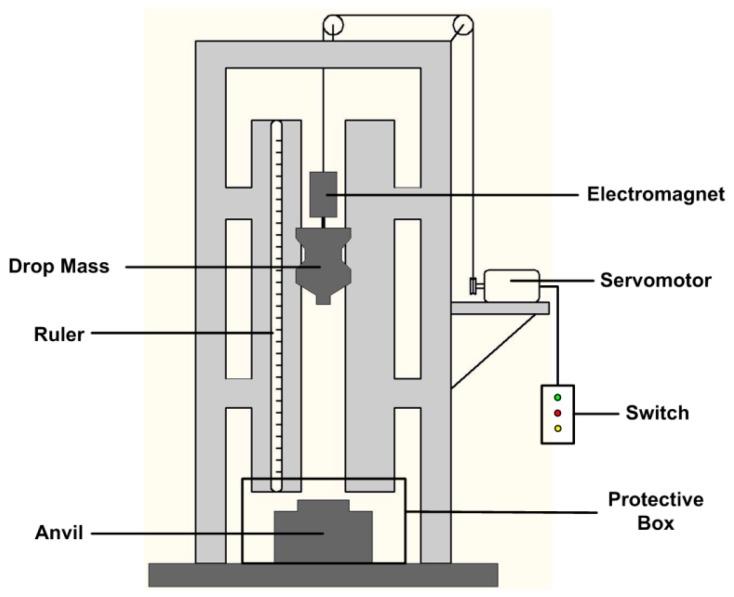
A schematic of the drop-weight apparatus used in this research.

As discussed in Section 1, a standard drop-weight apparatus was employed to investigate the sensitivity and impact-initiation characteristics of the materials, as illustrated shematically in Figure 2. The tester has a drop mass of 5.3 kg which can be released from a variable height in a range from 0 cm to 200 cm. The rise and release of the drop mass is controlled by an electromagnetic switch. The impact sensitivities of the three types of materials are compared by the characteristic drop height of impact sensitivity (H_50_), at which the material has a 50-percent probability of reaction. The test method by which the 50-percent point is obtained is an adaption of the well-known “up-and-down technique” [26]. In this research, a total of 16 tests were conducted for each of the types of materials. The dimensions of all the samples were Φ6 mm × 3 mm. Time sequences of the drop-weight tests were recorded by a Phantom V710 high-speed camera (Vision Research, Inc., Wayne, NJ, USA). During all the tests, a PMMA protective box was used to protect the safety of the researchers and the high-speed camera.

## 3. Results and Discussion

### 3.1. Mesoscale Characteristics

**Figure 3:**
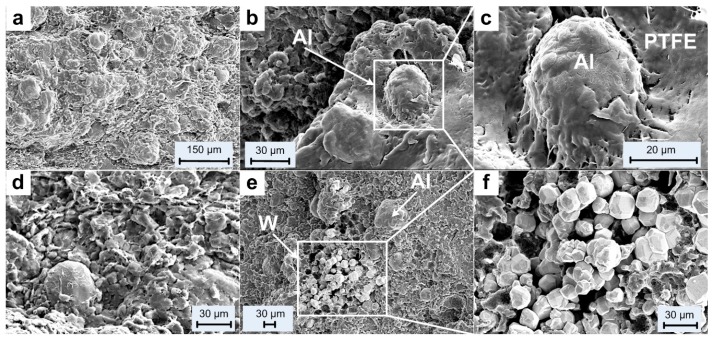
Microstructures of the PTFE/Al/W materials before tests: (**a**–**c**) type A material, with (**b**) and (**c**) showing an aluminum particle in the PTFE matrix; (**d**) type B material; (**e**,**f**) type C material, with (**e**) showing aluminum and tungsten particles and (**f**) showing aggregation of the tungsten particles.

Before the tests, the initial microstructures of the materials were characterized by a Scanning Electron Microscope (HITACHI S-4800, Tokyo, Japan), as shown in Figure 3.

Figure 3a,d illustrates the overall microstructural characteristics of the type A and type B materials. Metal particles can be distinguished easily by their spherical geometry from the surrounding matrix. PTFE matrix occurs as irregular flakes with flocculent edges in loose arrangement, and thus a lot of pores can be observed in the material structure.

An aluminum particle can be observed in Figure 3b and it is shown at a higher magnification in Figure 3c. The aluminum particle shows as an ellipsoid with its surface covered with PTFE. Obvious gap between the Al particle and the surrounding matrix can be observed while some places were connected by discontinuous thick PTFE fibers.

Figure 3e shows the distribution of the Al and W particles in type C material and the aggregation of the W particles are magnified in Figure 3f. The images show the W particles as brighter features than the Al particles because of their higher atomic number. Compared with the Al particles, the diameters of the W particles are much smaller. The W particles are loosely piled together and no combination with the matrix can be observed.

The main purpose of the sintering cycle of the PTFE/Al/W is to obtain cross-linking of the polymeric material and allow the particles to fuse together to form a homogeneous material. The root cause of the mechanical properties changes of the PTFE matrix composites is the change of the crystallinity of PTFE [27]. During the process of sintering, a series of physical and chemical changes happened, which is a reversible transformation process between the amorphous and crystalline state of the PTFE. The most widely applied fabrication process includes the stages of heating, temperature keeping and cooling. When the PTFE is heated above its melting point of 327 °C, the original crystalline structure would be destroyed while the material would become transparent and the volume would expand. Conversely, when the temperature drops below 327 °C, the PTFE would crystallize from the amorphous melt. Then, the volume of PTFE would shrink and it would become stiff and opaque again. Keeping the temperature at the highest level accelerates the movement of the molecules of PTFE. Particle/matrix interfaces between the particles would disappear and the material would become a compact and continuous whole. The crystallization proceeds most rapidly at around a temperature of 327 °C. Keeping the temperature at this level for several hours during the cooling process enables more complete crystallization and thus the strength, density and hardness of the PTFE matrix would be improved. Cooling rate after the temperature keeping stage also affects the recrystallization process. A lower cooling rate enables more sufficient recrystallization, whereas the decomposition of the PTFE will increase when the cooling rate is too low because it means that the PTFE will stay under high temperature for longer duration. Thus the strength of the material would decrease [28].

Compared with the sintering process employed by Zhang Xianfeng et al. [6], Liu Wang et al. [17] and Bin Feng et al. [22], the most obvious difference is the much lower sintering temperature, 300 °C. As a result, the crystallinity of the PTFE matrix remains almost the same as received. Then the matrix and the particles would not fuse together to form a homogeneous material, as described previously. As shown in Figure 3c,f, the gap between the aluminum particle and the matrix indicates an early debond at the interfacial area when loaded. The loosely piled up W particles would not improve the strength of materials at all. The slipping between particles would happen more easily among the W particles, and the shear failure of the matrix would happen there. Under these conditions, the materials would be more like they had been cold isostatic pressed, and the crystallinity of the matrix would not be the main influence of the mechanical properties.

### 3.2. Quasi-Static Compression Tests

**Figure 4:**
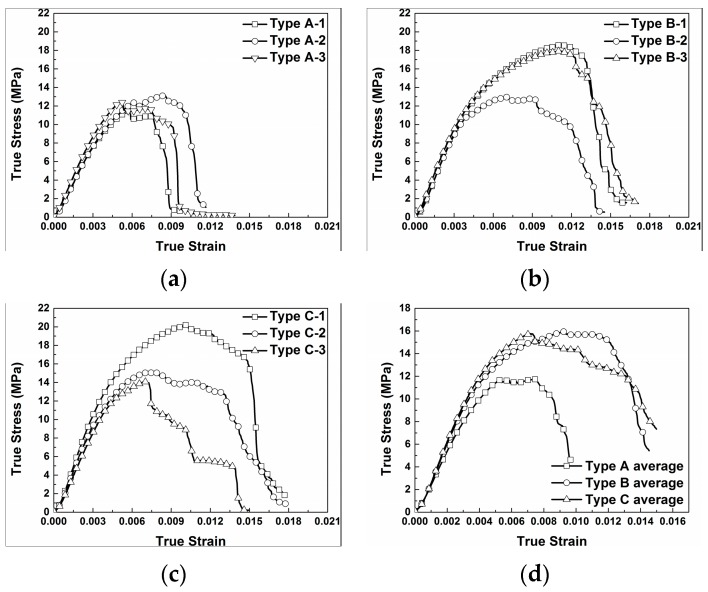
Quasi-static true stress-strain curves of PTFE/Al/W materials: (**a**) type A; (**b**) type B; (**c**) type C; (**d**) average stress-strain curves.

The true stress-strain curves of the PTFE/Al/W materials under quasi-static compression are presented in Figure 4. It can be observed that the type A material fails under a much lower stress and strain level than the other two types of materials. Meanwhile, the curves of the three types of materials show obvious deviation. Compared with the type A material, type B and type C materials have higher compressive strength, which indicates the reinforcing mechanism of the W particles. The curves of type B and type C composites show high scattering, while they have almost the same average compressive strengths, of 16.49 MPa and 16.46 MPa, respectively. However the type B composite has higher failure strain than type C, as can be observed in Figure 4d. This indicates that the mechanical property is not obviously enhanced with the increase of W particles addition but the plasticity decreases. This is because with the increase of W particles, the mechanical strength would be improved, on the one hand. However, on the other hand, the increased aggregation, resulting in a decrease of strength and plasticity would counteract the improvement of mechanical strength. For comparison, the compressive strengths and failure strains of all the samples were calculated. The compressive strengths were determined by the points where the samples started to fail on the stress-strain curves while the corresponding strains were the failure strains. The compressive strengths and failure strains from each test and their average values, as well as the coefficient of variation of the compressive strengths for each type of materials are tabulated in Table 2.

**Figure 5:**
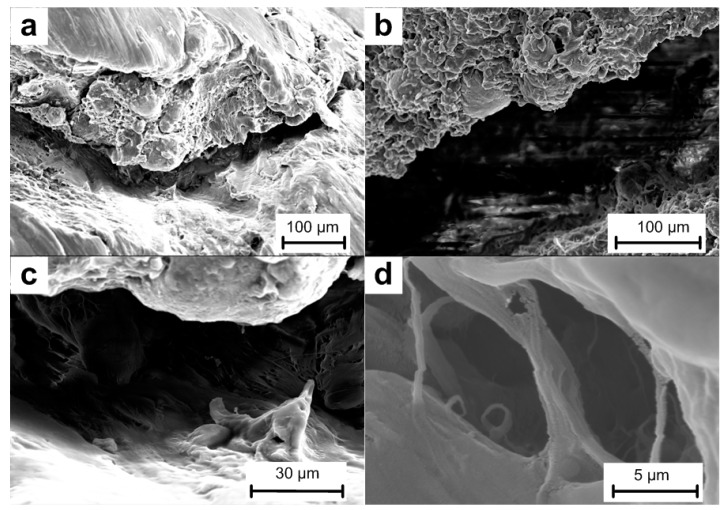
A sample of Material type B representing typical mesoscale characteristics of all test materials after quasi-static compression, shown at increasing magnification (**a**–**d**).

After quasi-static compression tests, a randomly selected crack on one of the recovered samples was characterized by the SEM under different magnifications, as shown in Figure 5. Observation of the fracture surfaces reveals three features: undeformed metal particles, smooth fracture surfaces and failure initiated from particle/matrix interfacial regions. Significantly different from the results obtained in the literature [4,17,29], nearly no PTFE fiber strings were observed between the fracture surfaces due to the mechanical loading. This should be attributed to the brittle property of the materials. The formation of matrix fibers during loading provides additional resistance for the propagating of cracks, and thus the strength would be improved. Another reason for the formation of the fibers is the temperature. The PTFE would form fibers when it is deformed at temperature above 30 °C [30]. However under quasi-static compression condition, nearly no heat was generated to lead to temperature rise, thus few fibers were formed.

### 3.3. Dynamic Compression Tests

**Figure 6:**
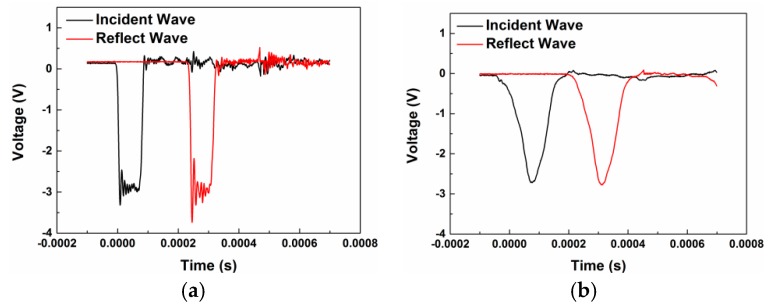
Incident and reflected wave (**a**) before and (**b**) after pulse shaping.

As described in Section 2.2, in order to minimize the effects of dispersion and achieve early dynamic stress equilibrium in the sample during the SHPB test, the incident pulse should be shaped. Figure 6 illustrates the incident and reflected waves before and after the pulse shaping by the polyurea shims placed between the striker and the incident bar. Before shaping, the incident and reflected waves were nearly square, with high frequency oscillations overlapped. After pulse shaping, ramp incident and reflected waves were achieved, while the high frequency oscillations were eliminated, which would help in minimizing the wave dispersion during compression. The rising edge of the incident wave was improved from ~15 μs to ~120 μs, which would ensure a higher pulse width and the early stress equilibrium within the samples.

**Figure 7:**
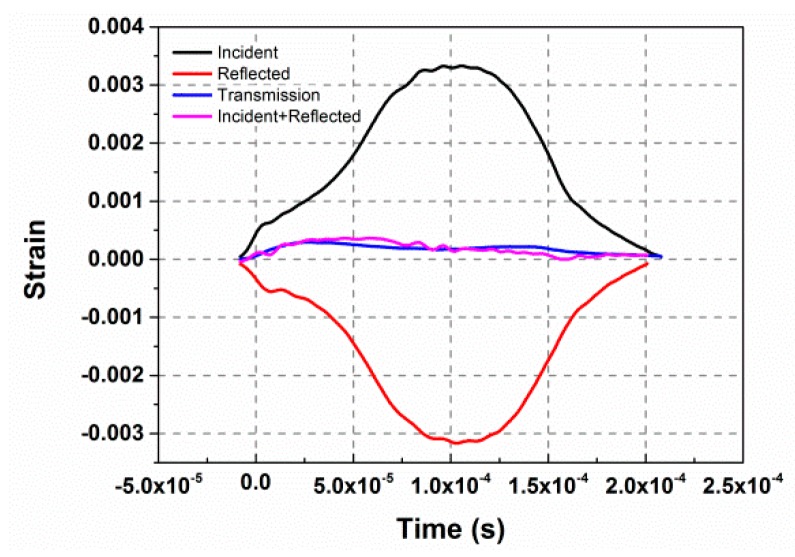
Comparison between the incident, reflected and transmission waves.

Stress-state equilibrium was verified by examining the one-wave stress determined from the transmitted pulse and the two-wave stress, determined from the incident and reflected pulses [31]. As can be observed from Figure 7, the transmission pulse and the superposition of the incident and reflected pulses show good consistency, which indicates a good stress equilibrium state within the sample.

**Figure 8:**
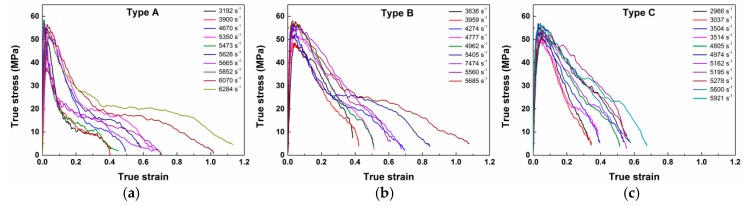
The true stress-strain curves of PTFE/Al/W at different strain rates: (**a**) type A; (**b**) type B; (**c**) type C.

Figure 8 presents the true stress-strain curves of the three types of materials under different strain rates. All the curves could be divided into two parts: rising nearly in a straight line with positive slope before the maximal point, demonstrating good linear elasticity at this strain stage, and dramatic decrease with negative slope after the maximal point. This indicates a brittle property for all the three types of PTFE/Al/W materials. Some of the curves have jagged characteristics near the highest points, due to the formation of micro cracks and the premature failure of the materials during loading. The first parts of the curves show only a small deviation among one another, indicating that the strain rate has no effect on the elastic parts. However, the slopes of the post-failure parts are closely related to the strain rates. The post-failure stage of the type A and type B materials consists of a first fast and a then slow decrease stage while that of the type C material only has a dramatic decrease stage, which indicates that the type C material is more brittle.

**Figure 9:**
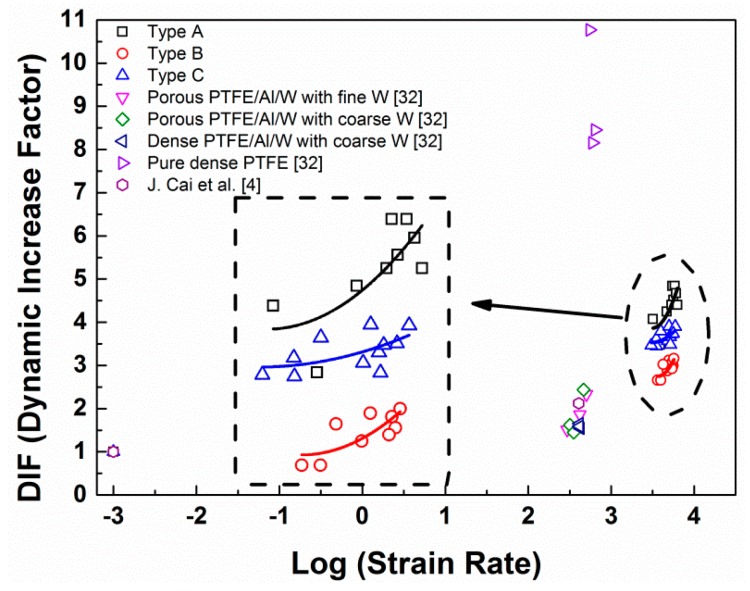
Strain-rate influence on the DIF of the PTFE/Al/W materials.

From the dynamic stress-strain curves, an apparent increase of the dynamic strength with the increase of the strain rates can be observed. The influence of loading strain rates on the dynamic compression strength increase of brittle materials can be described by the dynamic increase factor (DIF), which is defined by the ratio of the dynamic strength to the quasi-static strength in uniaxial compression, as defined by Equation (4):

(4) $$\mathrm{DIF}=\frac{f_{cd}}{f_{sd}}$$

where $f_{sd}$ and $f_{cd}$ are the uniaxial compressive strength in quasi-static and dynamic loading, respectively. Figure 9 shows the relationships between the logarithm values of strain rates and the DIFs of the PTFE/Al/W materials from our research and the references [4,32]. Among the three types of materials, the type A material has the highest DIFs, followed by the type C and B materials, which indicates that the type A material has the highest strain-rate sensitivity. The reason for this should be attributed to strength decrease induced through the introduction of W particles, and mesoscale imperfections. Apart from that of the pure dense PTFE, the DIFs of the materials studied in this research are higher than of the materials from the references. Generally the relationship between the logarithm strain rates and the DIFs can be described by a piecewise function because there exists a transition point of strain rate which divides the curve into high strain-rate sensitivity and low strain-rate sensitivity. In this research, the data points from the dynamic tests should be after the transition points and could be described by parabola-form curves, as indicated by the fitted curves in dashed box [33].

For type A:

$$\mathrm{DIF}=9.87{\left(log\dot{\varepsilon }\right)}^{2}-68.93log\dot{\varepsilon }+124.25$$

For type B:

$$\mathrm{DIF}=3.20{\left(log\dot{\varepsilon }\right)}^{2}-22.24log\dot{\varepsilon }+42.70$$

For type C:

$$\mathrm{DIF}=10.01{\left(log\dot{\varepsilon }\right)}^{2}-71.25log\dot{\varepsilon }+129.54$$

**Figure 10:**
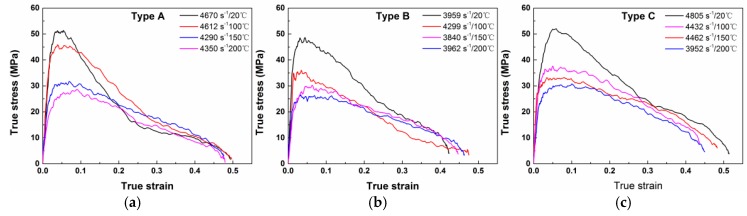
True stress-strain curves of PTFE/Al/W under different testing temperature: (**a**) type A; (**b**) type B; (**c**) type C.

The true stress-strain curves of the three types of PTFE/Al/W composites at elevated temperatures are presented in Figure 10. The curves show that the materials are strongly sensitive to environmental temperature at the same strain rate of ~4000 s^−1^: The initial modulus of elasticity and maximum failure stress are all dependent on environmental temperature. As the temperature rises, the stress-strain curves exhibit decreasing elastic modulus and maximum failure strength. The three types of materials are typical polymer matrix composites; therefore the above phenomenon should be attributed to the high temperature caused softening of the matrix. Same as the curves at room temperature in Figure 8, jagged characteristics can also be observed between the elastic stages and the maximal points. This phenomenon should be attributed to the same cause: the propagation of micro cracks and the premature failure of the materials during loading. The unloading portions of the three groups of materials show good consistency, due to the similar strain rates for all the curves, this indicates that the temperature has little effect on the unloading stage of the PTFE/Al/W composites. Table 3 tabulates the compressive strengths of the three types of materials at elevated temperatures and their decrease over the strengths at room temperature.

### 3.4. Drop-Weight Tests

**Figure 11:**
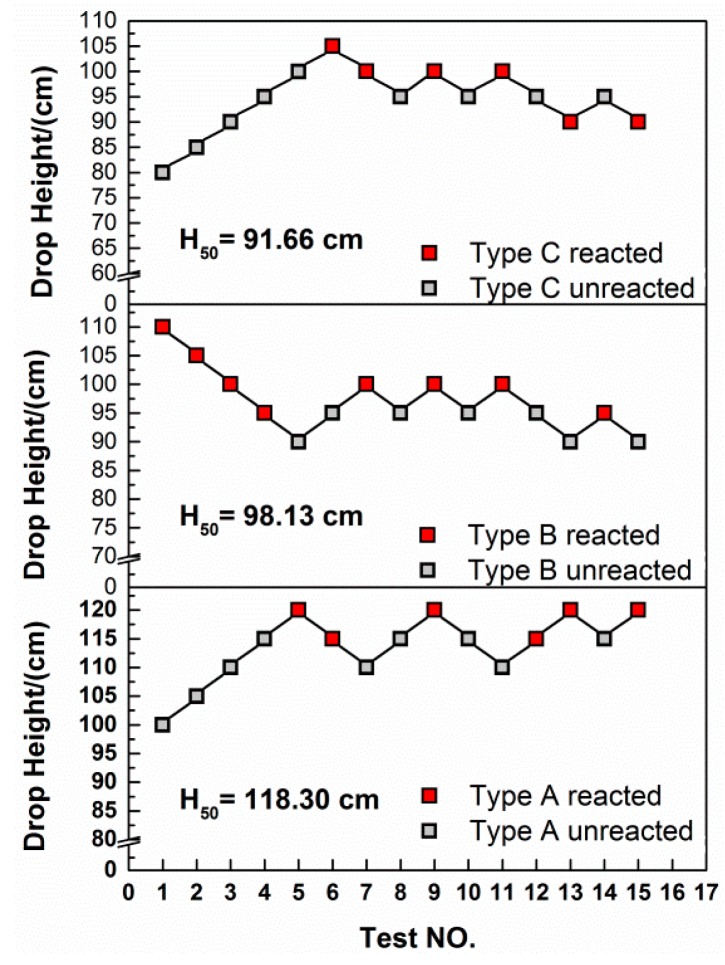
The drop-weight tests data points and the characteristic drop heights of impact sensitivity ($H_{50}$) for the three types of materials.

The impact sensitivities of the three types of materials are compared by the characteristic drop height of impact sensitivity ($H_{50}$), at which the material has a 50-percent probability of reaction. The test method by which the 50-percent point is obtained is an adaption of the well-known “up-and-down technique” [26]. In this research, a total of 16 tests were conducted for each of the types of materials. The $H_{50}$ is calculated by the following equation:

(5) $$H_{50}=\left[A+B\left[\frac{\sum \;iC_{i}}{D}-\frac{1}{2}\right]\right]$$

where *A* is the lowest height in the test, *B* is the increment of height, *D* is the number of reaction events among the tests, *i* is the order of the drop height starting from 0, $C_{i}$ is the number of reaction events under certain height. Experimental data points according to the “up-and-down” methods are recorded in Figure 11.

By Equation (5), the $H_{50}$ for type A, type B and type C materials are calculated as 118.30 cm, 98.13 cm and 91.66 cm, respectively, indicating that the type A material has the lowest sensitivity and with the increase of W content, the sensitivity of PTFE/Al material increases. It is not a common view because some researchers came to the opposite conclusion: the sensitivity would decrease with the increase of W content [34,35]. The W particles would not participate in the reaction between the PTFE matrix and Al particles, however with the addition of W particles, the materials become more brittle. Especially for the materials studied in this research, the aggregation of W particles makes the shear function and cracks more prone to form within the structure, which would result in the initiation of the reaction. Moreover, the extrusion and friction caused by the rigid W particles during loading would damage the oxide shell of aluminum particles to expose the aluminum core to surrounding oxidizers, which would also promote the initiation of the materials [36]. Though the quantity of reactant is decreased with the increase of W content, however, the maximum mass ratio of W is only 15 wt %, thus the effect of eliminating the initiation and reaction is not obvious.

**Figure 12:**
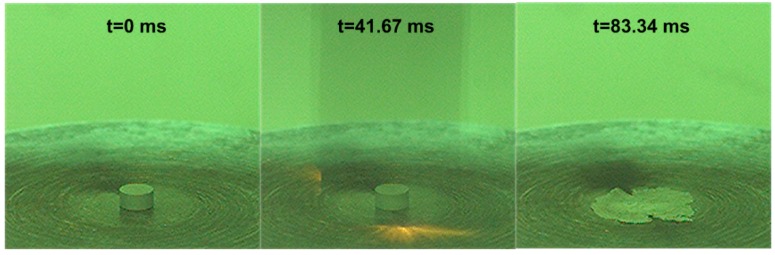
High-speed video sequences of the drop-weight tests.

**Figure 13:**
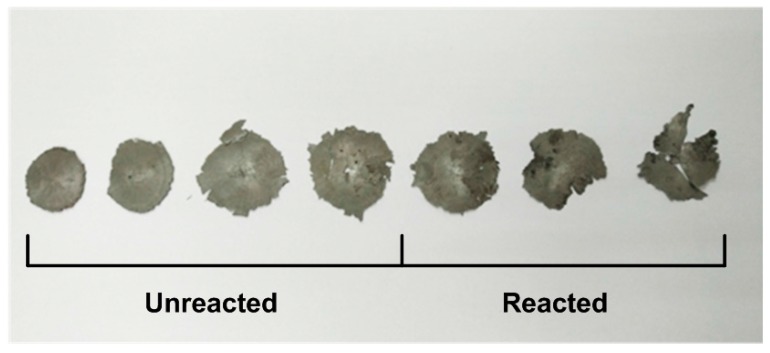
Recovered reacted and unreacted sample residues from drop-weight tests.

Figure 12 shows the high-speed video sequences of one of the drop-weight tests. In order to observe the sample during the test and the positions where the reaction initiated, we made a special case during the recording: the sample rate and exposure time were set as 24 fps and 39583.33 μs, which would provide a higher brightness but result in a blurred image of the drop mass in the second frame (*t* = 41.67 ms). Still, the injection of fire light and the black reaction product remaining on the anvil can be observed. In combination with the recovered reacted and unreacted sample residues in Figure 13, it can be observed that the reaction was initiated at two opposite positions near the edge of the samples. Before quenching, the reaction propagates from the edge to the center of the densified sample flake. This is because the vicinity of the outer surface of the cylindrical samples during compression is where the shear force concentrates the most intensively [20]. This indicates a shear-induced initiation mechanism, which is also proposed by other researchers [18]. Compared with the reacted samples, the unreacted were flattened into thin slices by the drop mass with uniform deformation around the edges.

## 4. Conclusions

Quasi-static compression, dynamic compression and drop-weight tests were conducted to study three types of PTFE/Al/W reactive materials fabricated under much lower sintering temperature which demonstrate brittle mechanical properties. By analyzing the experimental phenomenon and results, the following conclusions can be made:

The true stress-strain curves of the quasi-static tests show obvious deviations for each of the three types of materials. All the materials fail at very low strain and, by comparison, the type A composite has the lowest compressive strength which demonstrates the W particles reinforcement mechanism.

Mesoscale images of the samples before and after tests obtained by SEM indicate that the brittle property is mainly because of the low sintering temperature caused low crystallinity of the matrix and poor fusing together between the matrix and the metal particles. The other reason for the brittleness is the aggregation of the metal particles. Slipping between particles and early debonding at the interfacial regions are the main mechanisms for the early failure.

The true stress-strain curves of the materials at different strain rates consist of two parts: a short linear-elastic part and a dramatic decrease part. The compressive strength values are strain-rate sensitive. Also, the materials show a thermal softening phenomenon at elevated temperatures.

Characteristic drop heights of impact sensitivity ($H_{50}$) of the three types of materials show that the type C material has the highest sensitivity, indicating the increase in sensitivity with the increase of W content. The reason for this is that the addition and aggregation of W particles makes the shear function and cracks more prone to form and promotes the exposure of the aluminum core to surrounding oxidizers.

By analyzing the high-speed video sequences of the drop-weight tests and recovered sample residues, it is found that the reaction is initiated at two opposite positions near the edge of the samples where the shear force concentrates most intensively. This indicates a shear-induced initiation mechanism.

## Figures and Tables

**Table 1:** Compositions and theoretical material densities of the PTFE/Al/W granular composites.

Material Type	PTFE wt %	Al wt %	W wt %	Density (g/cm^3^)	TMD (g/cm^3^)
Type A	73.5	26.5	0	2.14	2.31
Type B	68.8	24.2	7	2.33	2.46
Type C	63.6	22.4	14	2.45	2.635

**Table 2:** Statistical results of the mechanical parameters of PTFE/Al/W under quasi-static compression and the coefficient of variation of the compressive strengths.

Materials	Compressive Strength (MPa)	Failure Strain	Average Compressive Strength (MPa)	Average Failure Strain	CoV (%)
Type A	11.48	0.00574	12.10	0.00574	2.59
	12.39	0.00627			
	12.44	0.00521			
Type B	18.60	0.01123	16.49	0.00979	1.78
	12.95	0.00703			
	17.94	0.01110			
Type C	20.20	0.01025	16.46	0.00836	3.64
	15.12	0.00782			
	14.06	0.00702			

**Table 3:** Compressive strengths of the PTFE/Al/W composites at elevated temperatures and the decrease over the strengths at 20 °C.

Temperature	Type A		Type B		Type C	
	Compressive Strength (MPa)	Decrease	Compressive Strength (MPa)	Decrease	Compressive Strength (MPa)	Decrease
20 °C	51.26	-	48.53	-	52.02	-
100 °C	45.87	10.52%	35.80	26.23%	37.58	14.44%
150 °C	31.67	38.22%	30.05	38.08%	33.12	36.33%
200 °C	28.39	44.62%	26.52	45.35%	30.78	40.83%
